# Secure-base leadership, job embeddedness, and intention to quit among Chinese physical education faculty

**DOI:** 10.3389/fpsyg.2026.1661925

**Published:** 2026-03-26

**Authors:** Shuguang Song, Yingliang Yang, Jingfeng Xu

**Affiliations:** 1 Faculty of Education, Qufu Normal University, Jining, China; 2 Department of Physical Education, China University of Petroleum (East China), Qingdao, China; 3 School of Information Engineering, Weifang Vocational College, Weifang, China

**Keywords:** Chinese higher education, intention to quit, job embeddedness, physical education faculty, secure-base leadership

## Abstract

**Background:**

Growing concerns about occupational health in academia underscore the importance of identifying relational and institutional resources that are associated with lower levels of educator disengagement and turnover-related withdrawal cognitions. Physical education faculty in Chinese higher education often face structural marginalization and limited institutional support, which may increase vulnerability to disengagement. Although leadership has been identified as a potential protective factor, the role of secure-base leadership in educational contexts remains underexplored.

**Purpose:**

This study examines whether secure-base leadership is positively associated with job embeddedness among Chinese physical education faculty and whether job embeddedness is associated with the relationship between leadership and intention to quit, conceptualized as a form of turnover-related withdrawal cognition. Job embeddedness is examined through its three dimensions—fit, links, and sacrifice—as a relational mechanism relevant to faculty retention.

**Methods:**

A three-wave panel design was employed with physical education faculty from Chinese universities. Secure-base leadership was measured at Time 1, job embeddedness at Time 2, and intention to quit at Time 3. Mediation analyses were conducted using PROCESS (Model 4), controlling for relevant demographic variables.

**Results:**

Secure-base leadership was negatively associated with intention to quit. This association was partially accounted for by all three dimensions of job embeddedness. Faculty perceiving higher levels of psychological safety and motivational support reported stronger institutional fit, more robust relational ties, and greater perceived costs associated with leaving.

**Implications:**

The findings highlight secure-base leadership as a relational resource that is positively associated with job embeddedness and inversely associated with early withdrawal cognitions among faculty in structurally vulnerable academic roles. Strengthening leader–faculty relationships may support retention processes and contribute to healthier, more sustainable academic environments in higher education.

## Introduction

Faculty retention is increasingly recognized as a cornerstone of occupational well-being and institutional sustainability in higher education, especially in disciplines such as physical education where professional marginalization, structural constraints, and lack of recognition may heighten educators’ vulnerability to disengagement and turnover-related withdrawal cognitions (i.e., intention to quit) (Böke and Norman, 2022). These risk factors, if left unaddressed, can contribute to broader patterns of educator ill-being and ultimately jeopardize student outcomes and program continuity. In the Chinese higher education context, these challenges are further shaped by socio-cultural norms that emphasize authority, harmony, and relational obligations, reinforcing the need for leadership practices that promote both individual resilience and organizational cohesion (Wang and Hu, 2017).

Secure-base leadership, grounded in attachment theory, offers a human-centered framework through which institutional leaders can respond to these risks. This approach fosters psychological safety, motivational support, and relational trust, thereby addressing core dimensions of educators’ occupational experience that are relevant to retention and wellbeing (London and Zobrist, 2024). By positioning leaders as emotionally available and supportive figures, secure-base leadership stands in contrast to traditional hierarchical models and may provide a critical buffer against early signs of occupational disengagement (Mayseless and Popper, 2019; Navas-Jiménez et al., 2024).

Although secure-base leadership has been examined in organizational and military contexts, its relevance in educational settings—particularly among physical education faculty—remains underexplored. This omission is notable, given that physical education instructors often face oversized classes, limited access to facilities, reduced institutional visibility, and professional isolation stemming from decentralized or peripheral teaching environments (Lei et al., 2023). These conditions, compounded by cultural pressures for conformity and deference, may undermine faculty morale and contribute to voluntary turnover.

At the same time, these characteristics make physical education faculty a theoretically informative group for studying how relational leadership and job embeddedness operate under conditions of professional marginalization and constrained resources. Focusing on a single discipline with relatively homogeneous work demands also reduces between-field variability and strengthens internal validity when examining the mechanisms linking leadership, embeddedness, and withdrawal cognitions.

Retaining committed and qualified physical education faculty is essential not only for program stability but also for the broader goals of promoting student health and fostering active lifestyles. Nevertheless, limited attention has been given to how relational leadership strategies might mitigate faculty withdrawal intentions and strengthen professional embeddedness. Job embeddedness—a construct encompassing employees’ perceptions of fit, social ties, and perceived sacrifices associated with leaving—has shown promise as a predictor of retention across sectors (Eslamlou et al., 2021; Mitchell et al., 2001; Ebeh et al., 2025; Zhao et al., 2025). Yet, its mediating role in educational contexts, particularly in relation to leadership, is still insufficiently understood (Lima, 2025). Although some contextual features of physical education (e.g., facility constraints, peripheral status within universities) are discipline-specific, the core psychological processes examined in this study—secure-base leadership, job embeddedness, and intention to quit—are not unique to this field. The mechanisms through which supportive leadership strengthens embeddedness and reduces withdrawal cognitions are conceptually applicable to other academic disciplines that face retention challenges, even if the concrete stressors and organizational arrangements differ.

This study seeks to address that gap by examining whether secure-base leadership reduces intention to quit among Chinese physical education faculty and whether this effect operates through enhanced job embeddedness. By identifying factors that support faculty connectedness and institutional commitment, the study contributes to ongoing efforts to understand and prevent early signals of occupational disengagement in education. The findings may inform institutional strategies that foster supportive leadership, strengthen professional integration, and ultimately contribute to a healthier and more resilient academic environment.

Although the general direction of the relationships among leadership, embeddedness, and withdrawal cognitions has been theoretically anticipated, empirical research has not previously tested secure-base leadership within Chinese higher education, nor examined how its effects unfold through the differentiated dimensions of job embeddedness across a multi-wave design. By integrating a relational leadership framework with a nuanced, multidimensional conceptualization of embeddedness, this study offers a theoretically meaningful advancement that moves beyond common-sense assumptions and clarifies the mechanisms through which supportive leadership reduces withdrawal cognitions.

At the same time, we acknowledge that the present focus on one disciplinary group sets boundaries on generalization. We therefore view this study as a discipline-specific test of theoretically broader mechanisms and encourage future research to examine whether similar patterns emerge among faculty in other fields and institutional contexts.

### Research gap and contributions

Despite the relevance of secure-base leadership for understanding relational dynamics in organizations, its application within higher education—and particularly within Chinese universities—remains theoretically underdeveloped. Existing scholarship has primarily examined secure-base leadership in Western corporate or military contexts, offering limited insight into how its core mechanisms of psychological safety, support, and exploration might operate under conditions shaped by hierarchical authority, relational obligation, and norms of harmony in Chinese academic environments. These cultural characteristics may intensify faculty dependence on supervisory relationships, making attachment-based leadership processes more salient than in Western settings and amplifying their relevance for understanding retention in disciplines marked by structural marginalization.

Furthermore, physical education faculty constitute a uniquely informative group for extending leadership theory. Their peripheral status within universities, restricted access to institutional resources, and heightened exposure to professional isolation suggest that relational leadership may function not only as a motivational resource but also as a compensatory relational asset in the context of limited structural support. This creates a theoretically meaningful boundary condition: secure-base leadership may be particularly relevant in professional groups whose work conditions make relational security especially consequential for well-being and retention.

The present study also advances the integration of attachment theory and conservation of resources (COR) theory by examining job embeddedness as a multidimensional mechanism associated with the relationship between secure-base leadership and withdrawal cognitions. Prior research in Chinese education has shown that teachers report turnover intentions under emotional labor demands, burnout, and institutional pressures, yet no studies have examined how leadership-related relational security is linked to job embeddedness or how embeddedness may function as a resource-based buffer in relation to intention to quit. Importantly, examining these processes across temporally separated measurement waves allows for a more rigorous assessment of their temporal ordering and reduces concerns associated with common method bias. By testing these associations in a multi-wave design and differentiating among the fit, links, and sacrifice dimensions of embeddedness, this study extends theoretical understanding of how attachment-informed leadership is related to resource accumulation processes that are relevant to early disengagement.

Overall, this research contributes novel theoretical value by (a) applying secure-base leadership to a professional group and cultural context in which its core mechanisms are especially salient, (b) identifying culturally shaped boundary conditions that inform how relational leadership is associated with embeddedness and withdrawal cognitions, and (c) offering the first empirical test linking secure-base leadership, the differentiated dimensions of job embeddedness, and intention to quit in Chinese higher education. These contributions move beyond contextual description to clarify theoretically meaningful relational and resource-based processes that deepen the explanatory potential of attachment-informed leadership perspectives.

### Theoretical framework and hypotheses development

#### Secure-base leadership and intention to quit

Secure-base leadership refers to a relational leadership style in which leaders create a sense of psychological safety while simultaneously encouraging autonomy and growth (Shaver and Mikulincer, 2014; Molero et al., 2019). Drawing on attachment theory, secure-base leaders function as reliable and emotionally available figures who provide stability, trust, and support, allowing followers to engage with their roles confidently while exploring opportunities for development. Unlike transactional or purely performance-oriented leadership models, secure-base leadership emphasizes enduring relational bonds between leaders and followers.

Although this leadership approach has received growing attention in business (Laguia et al., 2024) and defense contexts (Moriano et al., 2021), its application in higher education remains limited. Within the hierarchical and collectivist structure of Chinese universities, secure-base leadership may be particularly effective in reducing faculty members’ intention to quit by reinforcing emotional connection, relational security, and a sense of institutional belonging (Dong et al., 2025).

In the present study, intention to quit is conceptualized as a form of turnover-related withdrawal cognition—that is, the conscious consideration of leaving one’s position—rather than as actual turnover behavior. Such cognitions may emerge even in highly stable employment systems when teachers experience strain, reduced support, burnout, or a weakened sense of professional identity. Research conducted in China has consistently documented turnover-related withdrawal cognitions across educational levels, including kindergarten teachers (Li et al., 2025), primary and rural teachers (Zeng et al., 2024; Lu et al., 2025; Liu, 2022), and highly educated suburban and township teachers (Guo and Guo, 2024). Together, these findings indicate that structural employment stability does not preclude withdrawal cognitions but instead underscores their value as early indicators of diminished occupational well-being and organizational attachment.

From an attachment-based perspective, leadership relationships that fail to provide security and support may heighten uncertainty and disengagement, whereas secure-base leadership should reduce withdrawal cognitions by fostering trust, emotional safety, and confidence in the leader–institution relationship. Accordingly, faculty members who perceive higher levels of secure-base leadership are expected to be less likely to contemplate leaving their institutions.

*H1*: Secure-base leadership is negatively associated with intention to quit among Chinese physical education faculty.

#### Job embeddedness and secure-base leadership

Job embeddedness refers to the combination of forces that bind an employee to their role, including perceived fit with the institution, social and professional connections, and the anticipated costs of leaving (Huang et al., 2021). It provides a more comprehensive explanation for retention than traditional satisfaction or commitment models (Mitchell et al., 2001; Ng and Feldman, 2010; Peltokorpi et al., 2015). Recent bibliometric and content analyses further confirm job embeddedness as a central integrative framework for understanding why employees remain in their jobs across sectors and cultural contexts (Majumdarr and Dasgupta, 2024).

Leadership practices play a central role in shaping job embeddedness by influencing how employees experience alignment, connection, and investment within their institutions. Leaders can strengthen embeddedness by fostering interpersonal trust, aligning values and expectations, and facilitating professional integration (Sekiguchi et al., 2008). From an attachment-based perspective, secure-base leaders are particularly well positioned to enhance job embeddedness because they provide relational stability and emotional support while encouraging autonomy and growth. Such leadership behaviors are likely to reinforce employees’ sense of fit, deepen social and professional ties, and increase the perceived value of remaining within the organization.

*H2*: Secure-base leadership is positively associated with job embeddedness among Chinese physical education faculty.

#### Job embeddedness and intention to quit

Intention to quit—the conscious consideration of leaving one’s job—is a well-established antecedent of actual turnover behavior and a key indicator of early disengagement (Sender et al., 2018; Shah et al., 2020; Sun et al., 2012). In academic settings, elevated intention to quit is often interpreted as a signal of organizational strain, weakened attachment, and potential disruption to educational quality.

Research conducted in Chinese educational contexts similarly indicates that teachers who experience lower perceived fit, weaker social ties, or diminished professional identity are more likely to report stronger turnover-related withdrawal cognitions, even when actual job mobility is limited (Li et al., 2025; Zeng et al., 2024). These findings underscore that intention to quit reflects a meaningful psychological distancing from the institution rather than an immediate plan to resign.

Consistent with this view, job embeddedness functions as a protective factor against intention to quit by strengthening the personal, relational, and structural stakes associated with staying. Faculty members with higher levels of embeddedness are more likely to perceive congruence between their roles and values, maintain meaningful professional connections, and recognize the losses—both tangible and intangible—that would accompany departure (Zhang et al., 2019). As embeddedness increases, withdrawal cognitions are expected to diminish accordingly.

*H3*: Job embeddedness is negatively associated with intention to quit among Chinese physical education faculty.

#### The mediating role of job embeddedness

The relationship between leadership and withdrawal-related outcomes is rarely direct; rather, it unfolds through relational and contextual mechanisms that shape how employees experience their connection to the organization. Job embeddedness represents one such mechanism, capturing the extent to which individuals are integrated into their work environment through perceived fit, social ties, and accumulated investments (Hom et al., 2009; Karatepe and Shahriari, 2014).

From an attachment-based and resource-oriented perspective, secure-base leadership is expected to foster job embeddedness by providing relational security, reinforcing value alignment, and strengthening perceptions of mutual commitment between faculty and institution. By offering emotional availability and developmental support, secure-base leaders may initiate resource gains that enhance employees’ sense of belonging and investment, thereby reducing uncertainty and withdrawal-related cognitions. As embeddedness increases, the psychological and professional costs associated with leaving become more salient, lowering the likelihood that faculty members contemplate exit.

Accordingly, job embeddedness is conceptualized as a key mediating pathway through which secure-base leadership translates into reduced intention to quit among physical education faculty in Chinese higher education.

*H4*: Job embeddedness mediates the relationship between secure-base leadership and intention to quit among Chinese physical education faculty.

#### Summary of hypotheses

Based on the theoretical model described above, the following hypotheses are proposed:

*H1*: Secure-base leadership is negatively associated with intention to quit.

*H2*: Secure-base leadership is positively associated with job embeddedness.

*H3*: Job embeddedness is negatively associated with intention to quit.

*H4*: Job embeddedness mediates the relationship between secure-base leadership and intention to quit.

**Figure 1:**
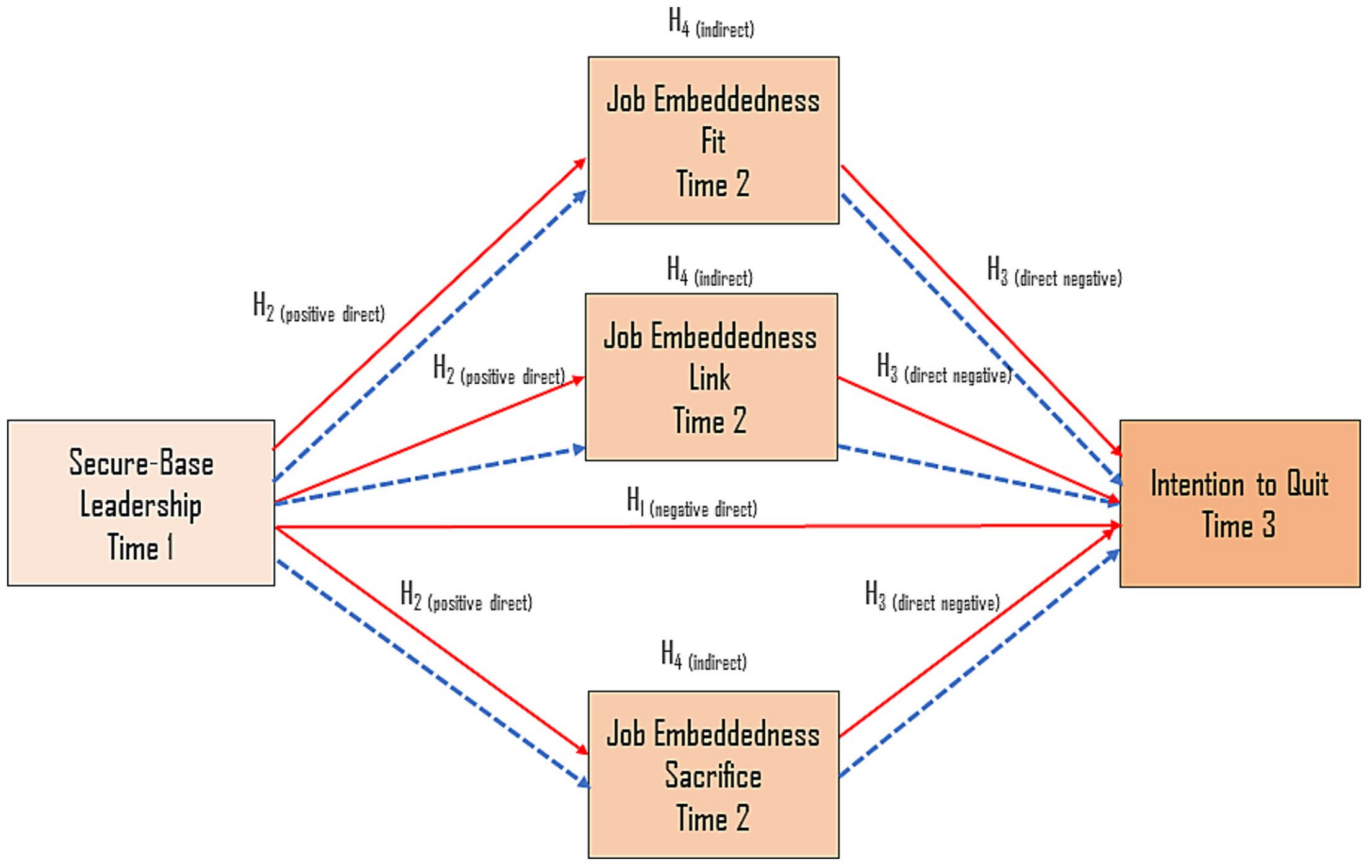
Full model of research with hypotheses. Solid arrows represent structural paths in the mediation model. Dotted arrows represent the corresponding indirect paths.

These hypotheses reflect the proposition that relational leadership enhances embeddedness, which in turn reduces the likelihood that faculty will consider leaving their institutions. The model focuses on the mediating role of job embeddedness as the core mechanism linking leadership to retention-oriented outcomes (Figure 1).

## Method

### Participants

The study included 851 physical education faculty members from various universities and colleges in China. The average age was 37.01 years (SD = 9.46), ranging from 24 to 51, indicating a broad distribution of ages among participants. On average, they had 7.15 years of professional experience (SD = 8.48), with a range from 0 to 41 years. Regarding educational background, 74.8% held a Master’s or Ph. D. degree, while 25.2% held only a Bachelor’s degree. Participants were employed across different types of institutions: 63.0% in public universities, 29.9% in private colleges, and 5.0% in foundation-funded institutions. A small proportion of data was missing in this category.

In terms of academic roles, 13.9% served in administrative or leadership positions such as department heads or course coordinators, 3.9% were identified as senior faculty with additional responsibilities, 16.7% were professors, and 57.5% were assistant professors. An additional 3.9% were classified in other professional roles, such as lecturers or research fellows. Concerning employment status, 83.3% of participants were working full-time, while 14.7% were employed part-time. The size of the academic units where participants worked varied: 25.1% were in departments with 1 to 9 employees, 44.7% in units with 10 to 49 employees, 14.0% in departments with 50 to 199 employees, and 16.2% in units with more than 200 employees.

Gender distribution was nearly equal, with 49.5% identifying as male and 50.5% as female. Most physical education faculty in Chinese universities are evaluated primarily on teaching, student training, and service responsibilities rather than research output. Consequently, strong publish-or-perish pressures—common in research-intensive disciplines—are not uniformly characteristic of this occupational group. For this reason, such pressures were not considered a structurally relevant factor in this study and were not included as a control variable.

### Procedure

This study was conducted with a sample of 851 physical education faculty members from higher education institutions across China, including public universities, private colleges, and foundation-funded institutions. Ethical approval was obtained from the Ethics Committee of Qufu Normal University on December 30, 2022 (Approval No. PSYHUM-24-28694.01). All procedures complied with institutional guidelines, national regulations, and the ethical principles of the Declaration of Helsinki (1964) and its subsequent amendments. Faculty were recruited through professional communication channels widely used among higher education staff in China, including discipline-specific WeChat groups, QQ academic communities, and online forums for physical education faculty. This approach resulted in a convenience-based sampling process with elements of snowball recruitment, which is appropriate for accessing a geographically dispersed professional group for whom no centralized sampling frame exists. Because the survey link circulated across numerous institutions and no institutional identifiers were collected, responses could not be tied to specific universities or departments. Consequently, no meaningful institutional-level clustering was identifiable in the dataset, and the distribution of responses across a broad range of institutions supports the assumption of independence of observations for the analyses conducted. Data were collected using Wenjuanxing (Questionnaire Star), a widely used and secure Chinese online survey platform that supports reliable large-scale questionnaire administration. Participation was voluntary, and all responses were anonymous. Prior to data collection, informed consent was obtained from all participants, and they were assured that their responses would remain confidential and used exclusively for research purposes. A self-generated identification code was used to match responses across waves while maintaining anonymity.

To minimize common method bias, a three-wave data collection procedure was implemented in line with the guidelines of Podsakoff et al. (2003). In Wave 1 (January 2025), participants completed demographic items (e.g., age, gender, academic qualification, tenure) and responded to the secure-base leadership scale. Of the 950 faculty members initially invited, 890 submitted usable questionnaires. Sixty participants were excluded due to nonresponse (*n* = 50) or invalid response patterns (*n* = 10). Wave 2 was conducted in April 2025, after a suitable time interval to reduce carryover effects and allow participants to reflect on their work experiences. In this wave, participants completed the job embeddedness measure, which assessed the dimensions of fit, links, and sacrifice. A total of 860 valid responses were received (96.6% retention from Wave 1), with 30 exclusions due to job changes, time conflicts, or incomplete responses. Wave 3 took place in May 2025 and focused on measuring intention to quit. At this stage, 851 faculty members completed the survey, forming the final sample used for analysis. Nine participants were excluded due to missing responses or transitions out of their current institutions.

### Instruments

#### Secure-base leadership (Time 1)

Secure-base leadership was measured using six items developed from the conceptual dimensions described by Kohlrieser et al. (2012) and from secure-base characteristics proposed by Feeney and Thrush. The items reflect three key aspects of secure-base leadership within a unified construct: (a) leader availability and emotional support during times of need (e.g., “My supervisor is sympathetic and supportive when I am worried or upset about something”; “My supervisor gives me encouragement and support when I have a difficult or stressful responsibility”); (b) the promotion of follower confidence and autonomy (“My supervisor encourages me to live up to my potential”; “My supervisor allows me to take an active role in setting my own performance goals”); and (c) encouragement of exploration (“When I tell my supervisor about something new that I would like to try, my supervisor encourages me to do it”).

All items were rated on a 5-point Likert scale (1 = strongly disagree, 5 = strongly agree). Secure-base leadership was modeled as a single latent construct, and reliability and validity were evaluated using composite reliability, average variance extracted (AVE), outer loadings, and HTMT within the PLS measurement model. The six items assessing secure-base leadership were adapted from the conceptual dimensions proposed by Kohlrieser et al. (2012) and secure-base characteristics identified by Feeney and Thrush. In the present study, these items formed a single latent construct with satisfactory psychometric properties. All indicators loaded significantly on the SBL factor (standardized loadings = 0.64–0.78, *p* < 0.001), and the construct demonstrated acceptable convergent validity (AVE = 0.50) and strong internal consistency (Cronbach’s *α* = 0.80; rho_A = 0.81; composite reliability = 0.86). A complete list of standardized outer loadings and confidence intervals is provided in Supplementary Tables S1.

#### Job embeddedness (Time 2)

Job embeddedness (Time 2) was assessed using the Chinese adaptation of the Global Job Embeddedness Items (GJEI) originally developed by Crossley et al. (2007) and translated by Zhang et al. (2012). The scale comprises three theoretically grounded dimensions: fit, links, and sacrifice. The fit dimension (four items) reflects the degree to which teachers perceive compatibility with their organization and community, as illustrated by items such as “I love the place where I live” and “I like the members of my workgroup.” The links dimension (five items) assesses the extent of teachers’ personal, organizational, and community connections, including indicators such as tenure in the position and family or housing ties. The sacrifice dimension (four items) gauges the anticipated losses associated with leaving one’s job or community, as reflected in statements such as “Leaving this community would be very hard.” Items employed either a 5-point Likert response scale or categorical formats consistent with the original GJEI. The full wording of all items used in this study appears in Supplementary Tables S2. Reliability and validity for each dimension were examined separately through composite reliability, AVE, outer loadings, and HTMT within the PLS framework.

#### Intention to quit (Time 3)

Intention to quit was measured using a four-item cognitive withdrawal scale derived from the classic turnover intention literature (Mobley, 1977; Cammann et al., 1979). The items capture thoughts about leaving one’s position, the evaluation of alternative employment opportunities, and anticipatory considerations regarding future tenure. Participants rated statements such as “I have considered resigning from my current position,” “I often think about leaving this institution,” and “I am likely to look for another job within the next year.” A fourth item (“I sometimes feel that I may not continue working at this institution in the future”) was included to assess anticipatory withdrawal cognitions. All items were rated on a 5-point Likert scale (1 = strongly disagree, 5 = strongly agree), and full item wording is available in the Supplementary materials. In line with contemporary psychometric standards, construct reliability and validity were evaluated using composite reliability, AVE, outer loadings, and HTMT in the PLS measurement model. Conceptually, intention to quit captures withdrawal cognitions rather than actual turnover behavior; such cognitions may arise even in employment systems characterized by high structural stability, including Chinese universities, and therefore serve as meaningful indicators of reduced institutional attachment and early signs of disengagement.

### Data analyses

To examine the relationships among secure-base leadership, the dimensions of job embeddedness (fit, links, and sacrifice), and Intention to quit, data analysis proceeded in several stages. First, descriptive statistics were computed to summarize the sample characteristics and central tendencies of the primary study variables. For each variable—secure-base leadership, job embeddedness fit, job embeddedness links, job embeddedness sacrifice, and Intention to quit—we calculated means, standard deviations, and minimum and maximum values to assess distributional properties and variability across the sample. Following this, Pearson correlation coefficients were used to explore the bivariate associations among the variables of interest. This preliminary step provided insight into the strength and direction of relationships between secure-base leadership, components of job embeddedness, and Intention to quit, thereby offering initial support for the hypothesized model.

To formally test the indirect effects proposed in the hypotheses, we conducted mediation analyses using PROCESS macro for SPSS, Model 4 (Hayes, 2022). Separate mediation models were estimated for each of the three job embeddedness dimensions—fit, links, and sacrifice—as parallel mediators in the relationship between secure-base leadership and Intention to quit. This analytical approach allowed us to assess not only the direct impact of secure-base leadership on Intention to quit, but also the extent to which this relationship was explained by each facet of job embeddedness.

Bootstrapping procedures were employed to evaluate the significance of the indirect effects. Specifically, 5,000 bootstrap samples were generated to estimate bias-corrected 95% confidence intervals for the indirect paths. The presence of mediation was determined by examining whether the lower-limit (LLCI) and upper-limit (ULCI) confidence intervals for each indirect effect excluded zero. Confidence intervals that did not contain zero were interpreted as evidence of statistically significant mediation. This resampling technique enhances the validity of inferences by addressing limitations related to non-normality and sample size, thereby increasing the robustness of the mediation findings.

## Results

### Assessment of common method variance

To evaluate potential common method variance, we conducted Harman’s single-factor test by entering all measurement items into an unrotated principal components analysis. The results showed that the first factor accounted for 31.30% of the total variance, well below the 50% threshold typically used to indicate substantial common method bias. Moreover, four components exhibited eigenvalues greater than 1, suggesting that no single factor dominated the covariance structure. These results indicate that common method variance is unlikely to pose a serious threat to the validity of the findings.

### Measurement model: convergent and discriminant validity

Before testing the structural relations, we evaluated the measurement model using PLS-SEM. Convergent validity was examined through standardized outer loadings, average variance extracted (AVE), and composite reliability. All indicators loaded significantly on their intended constructs (*p* < 0.001), with standardized loadings ranging from 0.64 to 0.83 and most exceeding 0.70. AVE values met the recommended 0.50 threshold for all constructs, ranging from 0.50 for secure-base leadership to 0.66 for intention to quit, indicating that each latent variable explained at least half of the variance in its indicators. Composite reliability coefficients were high and satisfactory, with rho_c values between 0.83 and 0.89 and rho_A values between 0.73 and 0.83. Cronbach’s alpha values fell within a similar range (0.73–0.83), supporting internal consistency for secure-base leadership, the three dimensions of job embeddedness (fit, links, sacrifice), and intention to quit.

Discriminant validity was assessed using the HTMT criterion and latent variable correlations. HTMT values ranged from 0.37 to 0.91 across construct pairs, and all 95% confidence intervals fell below 1.00, indicating that the constructs were empirically distinct despite their theoretical relatedness. The highest HTMT value was observed between the links and sacrifice dimensions of job embeddedness (HTMT = 0.91), which remains acceptable for closely related subdimensions of a broader construct. Latent factor correlations ranged from 0.33 to 0.70 in absolute value, further supporting discriminant validity.

Collinearity diagnostics indicated no issues with multicollinearity. Outer VIF values were consistently low (approximately 1.30–1.80), and inner VIF values for the mediators and the outcome ranged between 1.32 and 2.39, all within acceptable thresholds. Together, these results confirm that the measurement model demonstrates adequate convergent and discriminant validity and is suitable for subsequent structural analyses.

All measurement model evaluations were conducted in SmartPLS 4 using 5,000 bootstrap resamples. A complete list of standardized outer loadings, confidence intervals, and significance values for all indicators is provided in the Supplementary materials (Table 1).

**Table 1:** Convergent and discriminant validity of study constructs.

Construct	AVE	Composite reliability (ρc)	rho_A	Cronbach’s *α*	HTMT with other constructs
Secure-base leadership (T1)	0.50	0.86	0.81	0.80	JE–Fit: 0.43; JE–Links: 0.49; JE–Sacrifice: 0.61; ITQ: 0.37
Job embeddedness – fit (T2)	0.55	0.83	0.73	0.73	JE–Links: 0.81; JE–Sacrifice: 0.69; ITQ: 0.47
Job embeddedness – links (T2)	0.54	0.86	0.79	0.79	JE–Sacrifice: 0.91; ITQ: 0.46
Job embeddedness – sacrifice (T2)	0.58	0.85	0.77	0.76	ITQ: 0.47
Intention to quit (T3)	0.66	0.89	0.83	0.83	—

AVE = Average Variance Extracted. HTMT values represent heterotrait–monotrait ratios of correlations; all 95% confidence intervals were below 1.00. CR and rho_A values indicate satisfactory construct reliability. All outer loadings were significant at *p* < 0.001 and ranged from 0.64 to 0.83.

Descriptive statistics revealed meaningful variability across the study variables, reflecting diverse experiences among physical education faculty in terms of perceived leadership support, job embeddedness, and Intention to quit. Mean scores for secure-base leadership and the three dimensions of job embeddedness (fit, links, and sacrifice) were above the midpoint of the scale, whereas Intention to quit was notably lower, suggesting generally favorable perceptions and relatively low intent to leave among participants.

Pearson correlation analyses indicated statistically significant associations among all variables in the model. Secure-base leadership was positively correlated with all three dimensions of job embeddedness—fit (*r* = 0.319, *p* < 0.01), links (*r* = 0.371, *p* < 0.01), and sacrifice (*r* = 0.470, *p* < 0.01)—suggesting that faculty who perceived their supervisors as secure-base leaders also felt more connected to their roles and institutions. In contrast, secure-base leadership was negatively correlated with Intention to quit (*r* = −0.294, *p* < 0.01), indicating that higher perceived leadership support was associated with a reduced desire to leave.

All dimensions of job embeddedness were also negatively correlated with Intention to quit: fit (*r* = −0.371, *p* < 0.01), links (*r* = −0.386, *p* < 0.01), and sacrifice (*r* = −0.381, *p* < 0.01). These results provide initial empirical support for the proposed direct and indirect relationships among the constructs (see Table 2).

**Table 2:** Descriptive Statistics and Correlations.

Variable	M	SD	1	2	3	4	5
1. Secure-base leadership	3.74	0.46	--				
2. Job embeddedness fit	3.42	0.70	0.319**	--			
3. Job embeddedness link	3.20	1.03	0.371**	0.607**	--		
4. Job embeddedness sacrifice	3.31	1.13	0.470**	0.515**	0.708**	--	
5. Intention to quit	2.35	1.05	−0.294**	−0.371**	−0.386**	−0.381**	--

*N* = 851. M = Mean; SD = Standard Deviation. **p < 0.01.

### Regression and mediation analyses

#### Direct and joint predictive effects

Table 3 presents the results of the regression analysis including secure-base leadership and the three dimensions of job embeddedness as predictors of intention to quit. Secure-base leadership showed a significant negative effect on intention to quit (*B* = −0.2692, SE = 0.0767, *β* = −0.1227, *p* < 0.001), indicating that higher levels of perceived leadership support were associated with lower quitting intentions. Each job embeddedness dimension—fit, links, and sacrifice—was also negatively associated with intention to quit, with standardized coefficients of *β* = −0.1799, *β* = −0.1371, and *β* = −0.1332, respectively. These results provide support for Hypotheses 1 and 3.

**Table 3:** Regression analysis of secure-base leadership and job embeddedness dimensions on intention to quit.

Predictor	*B*	SE	*t*	*p*	LLCI	ULCI	*β*
Secure-base leadership	−0.2692	0.0767	−3.5122	0.0005	−0.4197	−0.1188	−0.1227
Job embeddedness–fit	−0.2710	0.0590	−4.5914	0.0000	−0.3869	−0.1552	−0.1799
Job embeddedness–links	−0.1397	0.0483	−2.8921	0.0039	−0.2344	−0.0449	−0.1371
Job embeddedness–sacrifice	−0.1240	0.0429	−2.8897	0.0040	−0.2082	−0.0398	−0.1332

N = 851. SE = Standard Error; LLCI = Lower Limit of the 95% Confidence Interval; ULCI = Upper Limit of the 95% Confidence Interval; β = Standardized Coefficient. Intention to quit is the outcome variable.

#### Mediating role of job embeddedness dimensions

Fit. Secure-base leadership was positively associated with job embeddedness – fit (*B* = 0.4653, SE = 0.0474, *β* = 0.3195, *t* = 9.81, *p* < 0.001, 95% CI [0.3722, 0.5583]). Fit, in turn, was negatively associated with intention to quit (*β* = −0.1799, *p* < 0.001). The indirect effect was significant (−0.1261, BootSE = 0.0339, 95% CI [−0.1977, −0.0624]), supporting H4 for this mediating path, as Table 4 shows.

**Table 4:** Total, direct, and indirect effects of secure-base leadership on intention to quit.

Effect type	*B*	SE	*t*	*p*	LLCI	ULCI	*β*
Total effect	−0.6444	0.0721	−8.9419	0.0000	−0.7859	−0.5030	−0.2937
Direct effect	−0.2692	0.0767	−3.5122	0.0005	−0.4197	−0.1188	−0.1227
Indirect effects							
Total indirect	−0.3752	0.0512	—	—	−0.4805	−0.2800	−0.1710
Through fit	−0.1261	0.0339	—	—	−0.1977	−0.0624	−0.0575
Through links	−0.1116	0.0433	—	—	−0.2002	−0.0303	−0.0509
Through sacrifice	−0.1375	0.0540	—	—	−0.2471	−0.0329	−0.0627

Bootstrap sample size = 5,000. Indirect effects are considered significant if the 95% confidence interval does not include zero. LLCI = Lower Limit Confidence Interval; ULCI = Upper Limit Confidence Interval; SE = Standard Error.

Links. Secure-base leadership also significantly predicted the links component (*B* = 0.7991, SE = 0.0687, *β* = 0.3709, *t* = 11.62, *p* < 0.001). Job embeddedness – links was negatively related to intention to quit (*β* = −0.1371, *p* = 0.0039). The corresponding indirect effect was significant (−0.1116, BootSE = 0.0433, 95% CI [−0.2002, −0.0303]).

Sacrifice. Leadership was also a strong predictor of job embeddedness – sacrifice (*B* = 1.1090, SE = 0.0715, *β* = 0.4702, *t* = 15.51, *p* < 0.001). Sacrifice negatively predicted intention to quit (*β* = −0.1332, *p* = 0.0040), and the indirect effect was significant (−0.1375, BootSE = 0.0540, 95% CI [−0.2471, −0.0329]).

Together, these results confirm that job embeddedness (fit, links, and sacrifice) partially mediates the effect of secure-base leadership on intention to quit.

#### Model fit and explained variance

The models demonstrated good explanatory power. Variance explained for each job embeddedness dimension was as follows:

Fit: *R*^2^ = 0.1021, MSE = 0.4438, *F*(1, 847) = 96.32, *p* < 0.001.

Links: *R*^2^ = 0.1376, MSE = 0.9332, *F*(1, 847) = 135.11, *p* < 0.001.

Sacrifice: *R*^2^ = 0.2211, MSE = 1.0099, *F*(1, 847) = 240.47, *p* < 0.001.

The overall model predicting intention to quit explained 20.6% of the variance [*R*^2^ = 0.2064, MSE = 0.8938, *F*(4, 844) = 54.88, *p* < 0.001]. The simpler model including only secure-base leadership explained 8.6% of the variance [*R*^2^ = 0.0863, MSE = 1.0255, *F*(1, 847) = 79.96, *p* < 0.001], highlighting the added explanatory value of job embeddedness, as Table 5 displays.

**Table 5:** Total effect of secure-base leadership on intention to quit.

Predictor	*B*	SE	*t*	*p*	LLCI	ULCI	*β*
Constant	4.7708	0.2723	17.5195	0.0000	4.2363	5.3053	—
Secure-base leadership	−0.6444	0.0721	−8.9419	0.0000	−0.7859	−0.5030	−0.2937

*N = 851. Total effect represents the unmediated impact of supervisor–subordinate Guanxi on intention to quit. LLCI = Lower Limit Confidence Interval; ULCI = Upper Limit Confidence Interval.*

**Figure 2:**
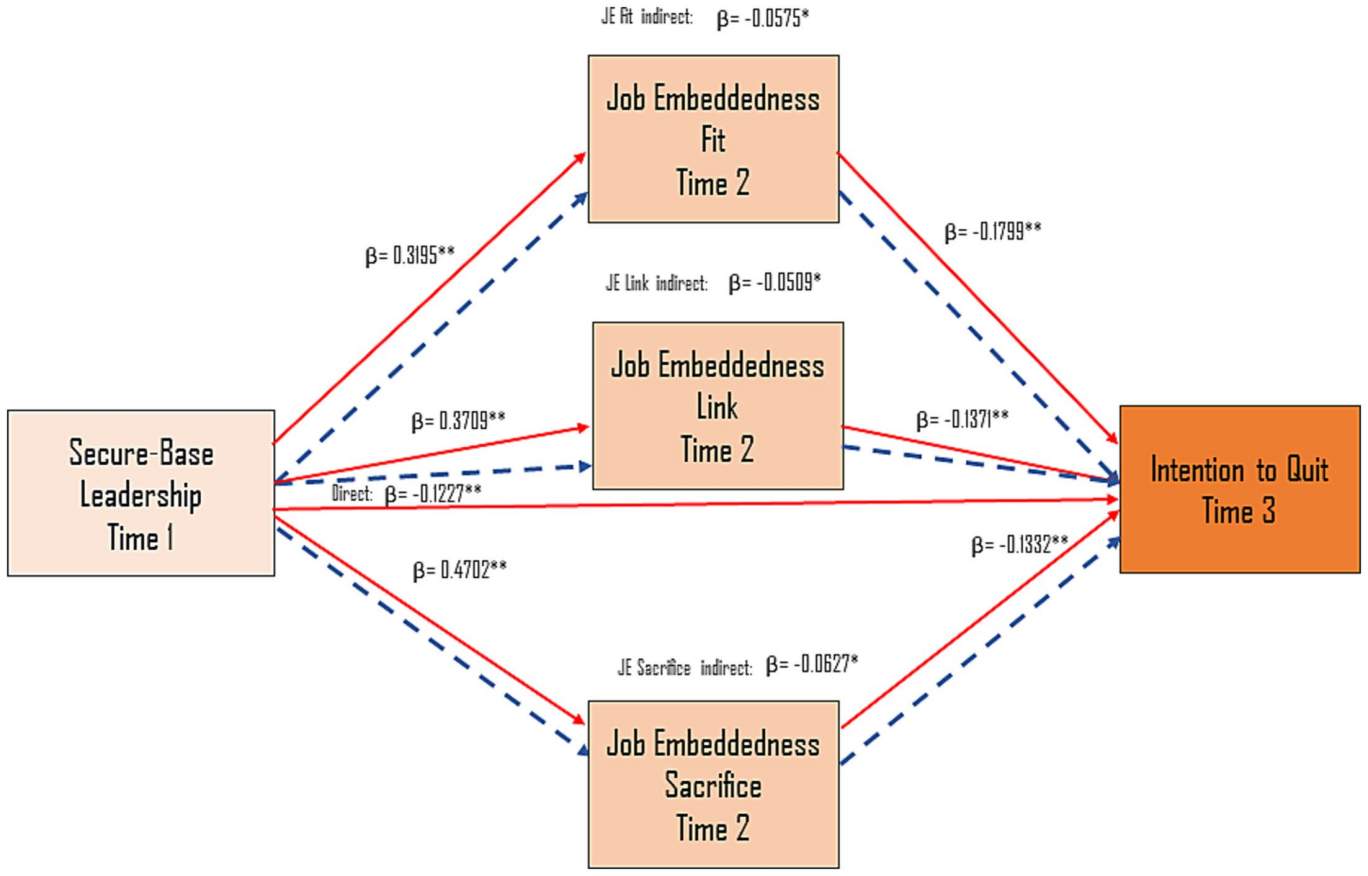
Standardized estimates for the regression model linking secure-base leadership, job embeddedness dimensions, and intention to quit. Solid arrows represent structural paths in the mediation model. Dotted arrows represent the corresponding indirect paths. Standardized coefficients (*β*) are shown with significance levels (*p < 0.05, ** *p <* 0.01, *** *p <* 0.001).

The standardized coefficients for all direct, indirect, and total paths are illustrated in Figure 2.

### Supplementary analyses

Given the potential relevance of employment status for withdrawal cognitions, we conducted additional analyses to examine whether the mediation model was consistent across full-time and part-time faculty. Employment status (0 = full-time, 1 = part-time) was first added as a covariate in the main model; its inclusion did not alter the direction or significance of the key relationships. We then re-estimated the mediation model separately for full-time (*n* = 695) and part-time (*n* = 123) faculty. Among full-time faculty, secure-base leadership predicted lower intention to quit both directly and indirectly through higher job embeddedness, mirroring the results of the full-sample analysis. Among part-time faculty, secure-base leadership was strongly associated with all three dimensions of embeddedness, and the total indirect effect remained significant, although some individual paths did not reach significance, likely due to reduced statistical power in this smaller subgroup. Full estimates for both subsamples are provided in Supplementary Tables S3.

## Discussion

### Direct effect of secure-base leadership on intention to quit

The findings indicate that secure-base leadership is significantly associated with lower levels of intention to quit among physical education faculty. Faculty who perceive their leaders as emotionally available, trustworthy, and motivationally supportive report substantially lower withdrawal cognitions, a pattern that is consistent with prior research linking relational leadership to psychological safety and institutional belonging (Navas-Jiménez et al., 2025; Bao, 2024). Rather than functioning merely as an interpersonal practice, secure-base leadership appears to operate as a relational resource associated with lower levels of early disengagement, particularly in settings where structural and professional vulnerabilities are salient.

The relevance of these findings becomes clearer when situated within the broader Chinese educational context. A growing body of research has documented that teachers across different educational levels report turnover intentions when exposed to emotional labor demands, organizational pressure, or insufficient support. For example, primary school physical education teachers exhibit higher intention to quit under elevated emotional labor demands (Qiu, 2023), and rural physical education teachers report withdrawal cognitions associated with burnout (Han, 2022). University faculty in private institutions similarly report turnover intentions linked to institutional pressure and heavy workloads (Wang, 2023), while university counselors show comparable patterns associated with organizational determinants (Zheng, 2022). Additional work with primary school teachers indicates that job stress is positively associated with withdrawal cognitions and that resilience partially buffers this association (Liu, 2022).

Collectively, these studies underscore that withdrawal cognitions are not incompatible with the structural stability of Chinese teaching positions. However, this literature has tended to emphasize risk factors rather than protective mechanisms. The present study extends this line of research by identifying secure-base leadership as a relational resource associated with lower withdrawal intentions. By situating an attachment-informed leadership construct within a culturally grounded context and focusing on physical education faculty—a group often positioned at the periphery of university structures—the findings help clarify the conditions under which relational leadership is linked to early turnover-related cognitions. In doing so, the study contributes to leadership theory by suggesting that resource-providing leadership behaviors may be particularly relevant in environments characterized by professional marginalization and constrained institutional visibility.

### Mediating role of job embeddedness

The findings provide consistent evidence that all three dimensions of job embeddedness—fit, links, and sacrifice—are associated with the relationship between secure-base leadership and intention to quit. Rather than functioning as independent or competing mechanisms, these dimensions appear to operate as complementary interpersonal and contextual resources that help account for the observed association between supportive leadership and lower withdrawal cognitions.

The fit dimension captures perceived congruence between faculty members’ values, competencies, and their institutional environment. Secure-base leadership is associated with higher perceived fit, potentially through its links to psychological safety, autonomy support, and validation of professional identity. This interpretation is consistent with prior research showing that relational leadership is related to self-efficacy, engagement, and congruence with organizational values (Ma and Li, 2023), and aligns with evidence that culturally embedded relational dynamics such as guanxi are associated with stronger perceptions of compatibility and long-term orientation (Guan and Frenkel, 2019).

The links component reflects the interpersonal and structural connections anchoring faculty to their academic community. Supportive leadership may be associated with stronger relational ties by fostering trust, encouraging collaboration, and increasing opportunities for meaningful interaction. Prior studies indicate that embedded social connections are associated with retention, effort, and performance (Rahimnia et al., 2019; Wheeler et al., 2012), and that perceptions of organizational support strengthen the relational foundations of embeddedness (Dirican and Erdil, 2022). Although some evidence suggests that links do not always function as a mediator of turnover-related outcomes (Ben-Meir et al., 2024), the present findings suggest that in disciplines characterized by organizational peripherality—such as physical education—relationally grounded leadership may be particularly relevant for strengthening this dimension of embeddedness.

The sacrifice dimension captures the perceived cost of leaving valued aspects of one’s role, such as autonomy, status, or supportive relationships. Secure-base leadership is associated with higher perceived investments, potentially by reinforcing faculty members’ confidence, validating their contributions, and expanding access to relational and professional resources. This interpretation aligns with prior work showing that intrinsic motivation, job crafting, and positive supervisor–employee relationships are linked to higher embeddedness and lower turnover intentions (Moon et al., 2020; Ramaite et al., 2022).

### Theoretical contribution: why job embeddedness is a particularly appropriate mediator for secure-base leadership

A central theoretical contribution of this study lies in the conceptual alignment between the interpersonal foundations of secure-base leadership and the interpersonal–contextual nature of job embeddedness. Secure-base leadership is rooted in attachment-related processes—perceptions of emotional availability, relational trust, and encouragement of exploration—and therefore operates primarily at the relational level of analysis. Unlike social-cognitive leadership theories that emphasize categorization or group identification processes, secure-base leadership is grounded in dyadic leader–follower interactions.

Job embeddedness, although often treated as a structural construct, is fundamentally interpersonal in its core components. Perceived fit reflects relational validation and value alignment; links capture the density and quality of social connections; and sacrifice reflects the perceived loss of relational, identity-based, or contextual resources. These characteristics position job embeddedness as a theoretically coherent mediator for understanding how attachment-informed leadership is associated with withdrawal cognitions.

By integrating these two frameworks, the present study contributes to leadership theory by suggesting that the pathways linking secure-base leadership to retention-related outcomes may be best understood through relational resource mechanisms rather than purely attitudinal or motivational mediators. This perspective highlights how supportive leadership is associated with expanded perceived resource pools (consistent with COR theory), reinforced relational attachments, and heightened perceptions of the costs of disengagement.

### Cultural extension of secure-base leadership: from western attachment theory to Chinese relational contexts

A further theoretical contribution of this study involves extending secure-base leadership—originally grounded in Western attachment theory—to the cultural context of Chinese higher education. Secure-base leadership draws on Bowlby’s attachment framework and its later organizational adaptations, emphasizing emotional availability, relational trust, and encouragement of exploration. Although these foundations emerged primarily within Western, individualistic societies, several core principles resonate strongly with relational values embedded in Chinese culture. The supervisor–subordinate dimension of guanxi, for example, shares key features with secure-base relationships by emphasizing loyalty, mutual obligation, and socioemotional support. Likewise, cultural norms related to face (mianzi) and social harmony (he) elevate expectations of relational sensitivity and leader attentiveness—conditions under which secure-base leadership processes may be particularly salient. By documenting these associations among Chinese physical education faculty, the present study illustrates how attachment-informed leadership models can be meaningfully extended to collectivistic contexts, thereby broadening the theoretical scope and cross-cultural relevance of secure-base leadership.

### Limitations of the present research

Despite the valuable contributions of this study, several limitations should be acknowledged to guide future research. Although this study does not constitute a fully longitudinal design in the strict sense—given that not all constructs were assessed repeatedly across all time points—the three-wave data collection strategy represents a substantial methodological advancement over the predominantly cross-sectional designs that characterize much of the existing literature. By temporally separating the measurement of secure-base leadership, job embeddedness, and intention to quit, the present design strengthens temporal precedence among the focal variables and substantially reduces concerns related to common method variance. Nevertheless, the correlational nature of the data does not permit strong causal inferences regarding the directionality of the observed relationships. At the same time, we acknowledge that truly dynamic processes cannot be fully captured without repeated assessments of all key constructs across multiple waves. Future research would therefore benefit from fully longitudinal designs that track changes in leadership perceptions, embeddedness, and withdrawal cognitions over time to more precisely model their reciprocal and evolving relationships.

Second, the geographic and cultural context of the sample presents limitations. Participants were physical education faculty from one region in China, which may constrain generalizability. Because leadership processes and embeddedness perceptions are culturally embedded, studies conducted in additional provinces or in cross-cultural settings are needed to validate and extend the present findings (Lobato et al., 2024).

Third, the exclusive reliance on self-reported data introduces the possibility of common method variance and social desirability effects. Although the time-lagged design mitigates part of this concern, future research should incorporate multi-source assessments—such as supervisor evaluations, peer reports, or behavioral retention indicators—to strengthen methodological rigor.

Fourth, even though validated instruments were used, measurement invariance cannot be fully assumed across academic disciplines. Physical education faculty may interpret certain items differently due to the unique characteristics of their professional routines, such as visibility of work tasks or access to institutional resources. Qualitative methods, including interviews or focus groups, could help verify the contextual appropriateness of these scales and enrich construct interpretation.

Fifth, the job-specific characteristics of physical education faculty may limit applicability to other academic populations. Their daily responsibilities, levels of autonomy, and patterns of interaction with students differ from those in more traditional academic fields. Similar considerations have been noted in prior work showing discipline-specific variability in faculty experiences and retention dynamics (Mäkelä et al., 2014). Future studies should examine whether the mediating pathways observed here generalize to departments with different structural demands or professional expectations.

Finally, while this study focused on secure-base leadership as a relational resource, other leadership styles and contextual variables (e.g., institutional climate, workload, or career stage) may interact with embeddedness and shape turnover intentions. Future models could test these moderators or integrate broader organizational factors to offer a more comprehensive account of faculty retention processes.

These limitations encourage cautious interpretation of the findings while providing a foundation for future research to extend and refine the conceptual model proposed in this study.

### Practical implications for academic leaders and career counselors

The findings of this study highlight several practical strategies that may help academic leaders and career counselors support faculty retention processes, particularly in disciplines such as physical education where structural constraints and professional marginalization are often associated with heightened withdrawal cognitions.

Develop secure-base leadership capacities within academic units.

Institutions may benefit from investing in leadership training that emphasizes emotional availability, respectful communication, and developmental support. Such behaviors are positively associated with psychological safety and relational trust—two interpersonal resources that have been shown to be inversely related to cognitive withdrawal and disengagement (Zettna et al., 2025).

Enhance career counseling and developmental support.

Establishing accessible, well-trained career counseling services may help faculty navigate promotion pathways, clarify long-term goals, and strengthen adaptability, a construct consistently associated with performance and job satisfaction in educational roles (Tanjung et al., 2025). Structured mentoring and consultation programs may further support these associations.

Strengthen the components of job embeddedness through institutional design.

Administrators may seek to foster person–organization fit by aligning institutional missions with faculty values, to facilitate interpersonal links through collaborative structures and community-building initiatives, and to clarify the professional and personal investments associated with remaining in the institution (Mohi Ud Din and Zhang, 2025). Together, these practices are associated with higher levels of job embeddedness, which in turn are linked to lower withdrawal cognitions.

Promote continuous and strategic professional growth.

Transparent career ladders, professional development opportunities, and participation in professional networks are associated with stronger perceptions of progression and identity continuity among faculty (Sott and Bender, 2025). These initiatives are also linked to higher perceived fit and sacrifice by reinforcing long-term professional investment. Engage institutional stakeholders in faculty development.

Faculty retention is not solely shaped by formal leadership roles. Involving peers, administrative staff, and community partners may contribute to a broader culture of support and shared responsibility for faculty well-being. These relational ecosystems are associated with stronger interpersonal links within the organization and complement the role of secure-base leadership.

Use digital platforms to expand access to support and resources.

Digital communication tools and academic networks may facilitate the dissemination of career-related information, peer collaboration, and ongoing professional guidance. Such platforms are associated with expanded perceptions of connection and resource availability, extending embeddedness beyond the immediate departmental context.

Taken together, these strategies point to ways in which academic leaders and career counselors may cultivate relationally rich and developmentally supportive environments that are positively associated with higher job embeddedness and lower intention to quit among physical education faculty.

## Conclusion

This study shows that secure-base leadership is significantly associated with lower levels of intention to quit among physical education faculty in China, both directly and through its associations with the three dimensions of job embeddedness—fit, links, and sacrifice. Faculty who perceive their leaders as emotionally available and motivationally supportive tend to report stronger alignment with their institutions, more robust relational ties, and a greater recognition of the personal and professional value of staying. Together, these relational and contextual factors are linked to lower levels of early withdrawal cognitions.

The findings underscore the importance of relationally grounded academic environments, particularly in cultural contexts that value social harmony, respect for authority, and relational obligation. At the same time, the results draw attention to the structural constraints often faced by physical education faculty, including heavy teaching loads, limited resources, and peripheral departmental status. Within such contexts, secure-base leadership appears as a critical relational resource that is positively associated with higher embeddedness and professional sustainability.

The study also advances theoretical understanding by clarifying how attachment-based leadership processes are related to embeddedness-based resource mechanisms, offering a more nuanced account of how relational leadership is connected to withdrawal intentions. Rather than operating solely through attitudinal pathways, secure-base leadership is associated with deeper organizational integration processes involving identity, belonging, and perceived investment.

Future research should examine the proposed associations across different cultural and disciplinary settings and employ fully longitudinal designs to capture the temporal unfolding of leadership, embeddedness, and withdrawal cognitions. Extending the model to include additional contextual or psychological moderators would further enrich understanding of faculty retention processes.

In sum, this study contributes to the literature on leadership and retention by demonstrating robust associations between secure-base leadership, job embeddedness, and intention to quit. Attention to these relational and contextual mechanisms may support faculty well-being and institutional stability, thereby contributing to the long-term sustainability of physical education programs and the broader mission of higher education.

## Data Availability

The datasets presented in this study can be found in online repositories. The names of the repository/repositories and accession number(s) can be found below: the data supporting this study’s findings are available from the following open access link: https://osf.io/amkrc/?view_only=79a1b2474c4f4b61b6a8fce706096c31.
