# Supplementary material for: Secure-base leadership, job embeddedness, and intention to quit among Chinese physical education faculty
Source: Front Psychol. 2026 Mar 26;17:1661925. doi: 10.3389/fpsyg.2026.1661925 (PMC13061666; doi:10.3389/fpsyg.2026.1661925)
Supplement: Supplementary file 1 [file Table_1.docx]

**Supplementary**

**Table S1. Standardized Outer Loadings of All Measurement Items**

| **Construct / Item** | **Loading** | **95% CI (Bootstrap)** | **p-value** |
| --- | --- | --- | --- |
| **Secure-Base Leadership (T1)** |  |  |  |
| SBL1 – “Supervisor supportive when worried/upset” | .780 | [.749, .809] | < .001 |
| SBL2 – “Supervisor encourages during difficult tasks” | .688 | [.640, .730] | < .001 |
| SBL3 – “Supervisor encourages potential” | .734 | [.694, .769] | < .001 |
| SBL4 – “Supervisor allows goal autonomy” | .718 | [.678, .753] | < .001 |
| SBL5 – “Supervisor encourages exploration” | .641 | [.587, .687] | < .001 |
| SBL6 – “Supervisor offers advice when needed” | .678 | [.629, .719] | < .001 |
| **Job Embeddedness – Fit (T2)** |  |  |  |
| JEfit1 | .750 | [.707, .785] | < .001 |
| JEfit2 | .747 | [.703, .782] | < .001 |
| JEfit3 | .727 | [.681, .766] | < .001 |
| JEfit4 | .749 | [.707, .784] | < .001 |
| **Job Embeddedness – Links (T2)** |  |  |  |
| JElink1 | .747 | [.706, .782] | < .001 |
| JElink2 | .734 | [.693, .769] | < .001 |
| JElink3 | .726 | [.683, .763] | < .001 |
| JElink4 | .733 | [.692, .768] | < .001 |
| JElink5 | .748 | [.708, .782] | < .001 |
| **Job Embeddedness – Sacrifice (T2)** |  |  |  |
| JEsac1 | .763 | [.728, .795] | < .001 |
| JEsac2 | .763 | [.727, .794] | < .001 |
| JEsac3 | .740 | [.701, .773] | < .001 |
| JEsac4 | .790 | [.759, .817] | < .001 |
| **Intention to Quit (T3)** |  |  |  |
| ITQ1 | .821 | [.795, .841] | < .001 |
| ITQ2 | .805 | [.775, .830] | < .001 |
| ITQ3 | .829 | [.802, .851] | < .001 |
| ITQ4 | .802 | [.772, .827] | < .001 |

**Note**. All outer loadings are standardized and derived from the PLS measurement model. Confidence intervals based on 5,000 bootstrap samples.

**Table S2. Full Item List for All Study Variables (Time 1–Time 3)**

**Secure-Base Leadership (Time 1)**

Leader availability and emotional support

1. My supervisor is sympathetic and supportive when I am worried or upset about something.
2. My supervisor gives me encouragement and support when I have a difficult or stressful responsibility.
3. My supervisor offers advice or assistance when I am dealing with a difficult task or problem.

Promotion of confidence and autonomy
4. My supervisor encourages me to live up to my potential.
5. My supervisor allows me to take an active role in setting my own performance goals.

Encouragement of exploration
6. When I tell my supervisor about something new that I would like to try, my supervisor encourages me to do it.

**Job Embeddedness (Time 2)**

**Fit – Community**
7. I love the place where I live.
8. The weather where I live is suitable for me.

**Fit – Organization**
9. I like the members of my workgroup.
10. My coworkers are similar to me.

**Links – Organization**
11. How long have you been in your present position?
12. How long have you worked for this company?

**Links – Community**
13. Are you currently married?
14. If you are married, does your spouse work outside the home?
15. Do you own the home you live in?

**Sacrifice – Organization**
16. I have a lot of freedom on this job to decide how to pursue my goals.
17. The perks of this job are outstanding.

**Sacrifice – Community**
18. Leaving this community would be very hard.
19. People respect me a lot in my community.

**Intention to Quit (Time 3)**

1. I have considered resigning from my current position.
2. I often think about leaving this institution.
3. I am likely to look for another job within the next year.
4. I sometimes feel that I may not continue working at this institution in the future.

**Note**. All constructs were modeled as **single latent variables** in the PLS structural equation model. Unless otherwise indicated, items used a 5-point Likert scale (1 = strongly disagree, 5 = strongly agree). Links-related items follow categorical or frequency formats consistent with the original GJEI.

**Table S3.** *Results of Mediation Models Estimated in the Full Sample, Full-Time Subsample, and Part-Time Subsample*

| **Model / Effect** | **Effect** | **SE** | **95% CI (LL – UL)** | **Significant?** |
| --- | --- | --- | --- | --- |
| **A. Full Sample (N = 835) – Covariate Included** |  |  |  |  |
| **Direct effect (SSGT1 → ToIT3)** | -0.293 | 0.081 | [-0.453, -0.133] | Yes |
| **Indirect effect (Total)** | -0.395 | 0.050 | [-0.493, -0.299] | Yes |
| Through Fit | -0.136 | 0.036 | [-0.207, -0.068] | Yes |
| Through Links | -0.114 | 0.047 | [-0.209, -0.025] | Yes |
| Through Sacrifice | -0.145 | 0.056 | [-0.257, -0.037] | Yes |
| **Total effect** | -0.687 | 0.075 | [-0.835, -0.539] | Yes |
| **Effect of covariate (T1employ → ToIT3)** | -0.064 | 0.063 | [-0.188, 0.060] | No |
| **B. Full-Time Faculty Only (n = 695)** |  |  |  |  |
| **Direct effect (SSGT1 → ToIT3)** | -0.360 | 0.090 | [-0.537, -0.183] | Yes |
| **Indirect effect (Total)** | -0.398 | 0.058 | [-0.515, -0.287] | Yes |
| Through Fit | -0.151 | 0.042 | [-0.236, -0.072] | Yes |
| Through Links | -0.142 | 0.051 | [-0.245, -0.046] | Yes |
| Through Sacrifice | -0.106 | 0.063 | [-0.231, 0.014] | No (trend) |
| **Total effect** | -0.758 | 0.082 | [-0.919, -0.598] | Yes |
| **C. Part-Time Faculty Only (n = 123)** |  |  |  |  |
| **Direct effect (SSGT1 → ToIT3)** | 0.023 | 0.205 | [-0.384, 0.429] | No |
| **Indirect effect (Total)** | -0.374 | 0.117 | [-0.633, -0.170] | Yes |
| Through Fit | -0.097 | 0.061 | [-0.228, 0.020] | No (trend) |
| Through Links | -0.037 | 0.114 | [-0.289, 0.172] | No |
| Through Sacrifice | -0.240 | 0.147 | [-0.562, 0.004] | No (borderline) |
| **Total effect** | -0.351 | 0.206 | [-0.759, 0.056] | No |

**Note**. Non-significant effects (95% CI including zero) are marked as “No”.

All indirect effects use percentile bootstrap CIs with 5,000 samples.
